# Renal Involvement in Leptospirosis: The Effect of Glycolipoprotein on Renal Water Absorption

**DOI:** 10.1371/journal.pone.0037625

**Published:** 2012-06-06

**Authors:** Katia Regina Cesar, Eliete Caló Romero, Ana Carolina de Bragança, Roberta Morozetti Blanco, Patrícia Antonia Estima Abreu, Antonio José Magaldi

**Affiliations:** 1 Basic Research Lab-LIM 12, Nephrology-HCFMUSP, São Paulo, São Paulo, Brazil; 2 Bacteriology, Instituto Adolfo Lutz, São Paulo, São Paulo, Brazil; 3 Bacteriology Laboratory, Instituto Butantan, São Paulo, São Paulo, Brazil; Universidade de Sao Paulo, Brazil

## Abstract

**Background:**

Leptospirotic renal lesions frequently produce a polyuric form of acute kidney injury with a urinary concentration defect. Our study investigated a possible effect of the glycolipoprotein, (GLPc) extracted from *L. interrogans,* on vasopressin (Vp) action in the guinea pig inner medullary collecting duct (IMCD).

**Methods:**

The osmotic water permeability (Pf µm/s) was measured by the microperfusion *in vitro* technique. AQP2 protein abundance was determined by Western Blot. Three groups were established for study as follows: Group I, IMCD from normal (ngp, n = 5) and from leptospirotic guinea-pigs (lgp-infected with *L. interrogans serovar* Copenhageni, GLPc, n = 5); Group II, IMCD from normal guinea-pigs in the presence of GLPc (GLPc group, n = 54); Group III, IMCD from injected animals with GLPc ip (n = 8).

**Results:**

In Group I, Pfs were: ngp- 61.8±22.1 and lgp- 8.8±12.4, p<0.01 and the urinary osmolalities were: lgp-735±64 mOsm/Kg and ngp- 1,632±120 mOsm/Kg. The lgp BUN was higher (176±36 mg%) than the ngp (56±9 mg%). In Group II, the Pf was measured under GLPc (250 µg/ml) applied directly to the bath solution of the microperfused normal guinea-pig IMCDs. GLPc blocked Vp (200 pg/ml,n = 5) action, did not block cAMP (10^−4^ M,) and Forskolin (Fors- 10^−9^ M) action, but partially blocked Cholera Toxin (ChT- 10^−9^ M) action. GLP from *L.biflexa serovar patoc* (GLPp, non pathogenic, 250 µg) did not alter Vp action. In Group III, GLPc (250 µg) injected intraperitoneally produced a decrease of about 20% in IMCD Aquaporin 2 expression.

**Conclusion:**

The IMCD Pf decrease caused by GLP is evidence, at least in part, towards explaining the urinary concentrating incapacity observed in infected guinea-pigs.

## Introduction

Leptospirosis, caused by *Leptospira* spp infection, is one of the most common anthropozoonoses in the world [1]. It is cause for widespread alarm as it is currently an emerging disease with a high incidence in tropical countries and thus considered to be a major global veterinary and human health problem. Direct or indirect contact with *Leptospira*, via a contaminated environment, especially water polluted with urine from infected animals, or via infected animals, causes an infection characterized from mild symptoms (anicteric form) to severe infection [1]. The Weil’s syndrome, the most severe form of leptospirosis, is a classical model of sepsis that includes acute respiratory distress syndrome and acute renal injury [2], [3]. A great number of possible virulent factors such as hemolysin, flagellin, heat shock protein, outer membrane proteins (OMPs), lipopolysaccharides (LPS), glycolipoproteins (GLP) and others have been thought to be accountable for the infection, but to date, their effective role in leptospirosis pathogenesis is still unclear.

The renal injury observed in leptospirosis frequently produces a polyuric form of acute renal injury, followed by hypokalemia with an elevated urinary fractional excretion of potassium and an inability to concentrate urine. This increased kaliuresis is due to a decrease of potassium reabsorption in the damaged proximal tubule and to an increase of its secretion in the distal tubules [4], [5], [6,]. The concentrating defect is due, at least in part, to a decrease in the water permeability in the inner medullary collecting duct (IMCD) probably produced by a bacterial endotoxin [7]. One possible endotoxin that showed cytotoxic effects was identified as a glycolipoprotein extracted from *Leptospira* [8].

The purpose of this study was to investigate the effect of the GLPc in IMCD water absorption in normal and leptospirotic guinea pigs by different techniques. Guinea pigs were used as the experimental animals. Our results evidenced that the decrease of renal water absorption as a result of GLP action on the Vp receptor-Protein G complex should be taken into account as a causal factor contributing to the urinary concentration defect observed in leptospirotic non-oliguric Acute Kidney Injury (AKI).

## Materials and Methods

Normal male guinea pigs, weighing 170–180 g, were used. They were maintained under standard laboratory conditions and fed a normal pellet diet and tap water *ad libitum*. This rodent species was used because rats do not sicken after leptospira infection.

### A - Groups of Experiments

#### Group I - leptospirotic animals

During a one-week study period, all animals were fed standard chow with *ad libitum* access to tap water. The guinea pigs were infected by intraperitoneal inoculation with 1 ml of blood from an animal infected with *L interrogans serovar* Copenhageni [9]. The guinea pigs were placed in metabolic cages, five to six days after inoculation, when the jaundice was evident. Urinary volume and urinary osmolality, together with Blood Urea Nitrogen (BUN), were measured. After this period, the guinea pigs’ kidneys were removed to isolate the IMCD in order to measure the Pf by microperfusion *in vitro*.

#### Group II - normal animals

In this group, normal IMCDs were microperfused in contact with substances that can elicit the Vp cascade in different steps and in the presence of glycolipoprotein (GLP) extracted from Leptospira. The following substances used were: a) cycle Adenosine Mono Phosphate (cAMP), b) Forskolin and c) Cholera Toxin.

#### Group III - GLP animals

Normal guinea pigs were injected intraperitoneally with GLP in order to study its effect on Aquaporin 2 (AQP2) expression in IMCDs. After 12 hs, the guinea pigs were sacrificed and their kidneys were prepared in order to quantify the AQP2 expression. Guinea pigs without inoculation were used as control animals.

### B - Methodologies Used

Isolated Microperfusion *in vitro*. The isolated IMCD was perfused by the well-known *in vitro* microperfusion technique, previously described in the literature, in order to determine the Osmotic Water Permeability (Pf µm/s) [10], [11]. In brief, after pentobarbital (40 mg/kg body weight) anesthesia, tubules from the last one-third segment of renal papilla were isolated, immersed in a dish of chilled Ringer-HCO_3_ buffer, oxygenated, and kept at a pH of 7.4 by bubbling the solution with 5% CO_2_–95% O_2_. After isolation, the segment was transferred to a temperature-regulated chamber (37°C) and mounted onto the stage of an inverted microscope. To measure the net water absorption (*J*_v_), [^14^C] inulin, dialyzed immediately before the experiments, was used, and then added to the perfusion solution to reach a range of radioactivity of about 25–100 cpm/nl [11]. Data for each period are the average of three to four collections. According to this technique, the tubules remain in contact with the drugs for at least 40 minutes in each period before sample collection. After finishing each period, the chamber is washed with the bath solution and subsequently, a new bath solution containing another drug is added to initiate the new period. The mean perfusion rate, Vi (volume infused/min), was 30.9±0.08 nl/min, and the mean area of the tubules was 15.7±1.0×10^−4^ cm^2^.

#### Glycolipoprotein extraction

The procedure was carried out as described by Vinh et al [12]. *Leptospira biflexa* serovar Patoc strain Patoc I and *Leptospira interrogans* serovar Copenhageni strain M20 were cultured at 28°C in 1000 mL of liquid EMJH medium (Difco). The cultures were centrifuged at 18,000 g for 50 min at 4°C. The sediments were re-suspended in a solution of 0.01 mol l–1 Tris–HCl buffer (pH 7.4) containing lysozyme (50 *u*g ml^−1^) and kept overnight at 36°C. The lysozyme-treated leptospiras were centrifuged at 20,000 g for 30 min, and the supernatants were collected and treated with 50 µg ml^−1^ each of RNAse and DNAse (Sigma) for 3 h at 37°C, dialyzed against 0.01 mol 1^−1^ Tris-HCl buffer (pH 7.4) for 24 h at 4°C and finally acidified with 1 mol l^−1^ acetic acid to pH 3.7 at 4°C. The precipitated GLP was centrifuged at 20,000 g for 1 h, washed twice with 0.1 mol^−1^ acetic acid, re-suspended in distilled water (100 µl) and stocked at 4°C. Protein concentrations were quantified by Bradford [13] using Bio-Rad Protein Assay kit II (Boehringer, Ingelheim, Germany). GLPc and GLPp were obtained from the two strains, *L. interrogans* and *L. patoc*, respectively.

#### Aquaporin 2 expression

In order to study the effect of GLP on Aquaporin 2 (AQP2) expression in IMCDs in a group of animals (n = 5), 250 µg of this endotoxin were injected intraperitoneally. The guinea pigs were sacrificed and their kidneys were prepared to quantify the AQP2 expression, as explained below.

#### Western blot: preparation of membrane fractions

IMCD samples were isolated from the renal papilla and homogenized in cold solution (20 mM Mannitol, 80 mM Hepes, 41 mM KOH, pH 7.5) containing protease inhibitors (cocktail protease inhibitor, Sigma Chemical, St. Louis, MO, USA) using a Teflon pestle glass homogenizer (Schmidt and Co, Frankfurt/M, Germany) [14]. The homogenates were centrifuged at low speed (4000 g) for 15 min at 4°C in order to remove the nuclei and cell debris. Subsequently, the supernatants were centrifuged at 200,000 g for 1 h at 4°C (rotor 50Ti; Beckman Instruments, Palo Alto, CA, USA) to produce a pellet containing membrane fractions enriched with plasma membranes and intracellular vesicles. Protein concentration was determined for each sample using the Bradford method [13] (Bio-Rad Laboratories, Richmond, CA, USA).

#### Electrophoresis and immunoblotting

The proteins were separated on denaturing SDS 12% polyacrylamide gel by electrophoresis [14]. These proteins were then transferred to a polyvinylidene difluoride (PVDF) membrane by wet electroblotting for 90 min. Blots were blocked for 60 min at 4°C with 5% nonfat dry milk in PBS-T, pH 7.5 (phosphate-buffered saline, in mM: 100 NaCl, 80 Na_2_HPO_4_, 20 NaH_2_PO_4_, 0.1% Tween 20). Blots were incubated overnight with AQP2 antibody (1∶10,000 dilution) and with the control actin antibody (1∶2,000) and then washed and incubated with the second antibody (anti-goat secondary antibody HRP-conjugated, diluted at 1∶10,000) for 1 h. Subsequent detection of the specific proteins was carried out by enhanced chemiluminescence (ECL- Amersham), in accordance with the manufacturer’s instructions. Pre-stained protein markers (Sigma Chemical Co) were used for molecular weight determinations.

#### Quantification of AQP2 kidney levels

Enhanced chemiluminescence films with bands within the linear range were scanned using the Image Master VDS (USA). For AQP2, both the 29 kDa and the 35–50 kDa bands (corresponding to two different states of glycosylation) were quantified by densitometric analysis. Densitometry results are reported as integrated values (area x density of the band) and expressed in percentages when compared with the control actin protein abundance (100%).

#### Statistics

Differences among the means of multiple parameters were analyzed by ANOVA, followed by the Student-Newman-Keuls test. Differences between two parameters were analyzed either by paired *t*-test or nonparametric methods (Mann-Whitney test). Values of p<0.05 were considered significant.

#### Chemicals

The isotopic materials used were bought from Amersham International and New England Nuclear. GLPs were extracted in the Leptospirosis Sector of the Center of Bacteriology at Adolfo Lutz Institute, São Paulo, Brazil. The other drugs were purchased from Sigma Chemical, Saint Louis, MO, USA, Santa Cruz Biotechnology, Santa Cruz, CA, USA, Amersham Biosciences, Sweden, and Pharmacia Biotech, Uppsala, Sweden.

#### Ethics statement

The present study was approved by the ethical commissions of the University of São Paulo Medical School, the Adolfo Lutz Institute and the Butantan Institute. The animals were handled in accordance with the standard for good animal practice as defined by the Brazilian code of practice for the care and use of animals for scientific purposes (Law no 11794, 10/08/2008) and regulated by the National Council of Animal Experimentation Control- CONCEA.

## Results

### Group I - Leptospirotic Animals

**Figure 1:**
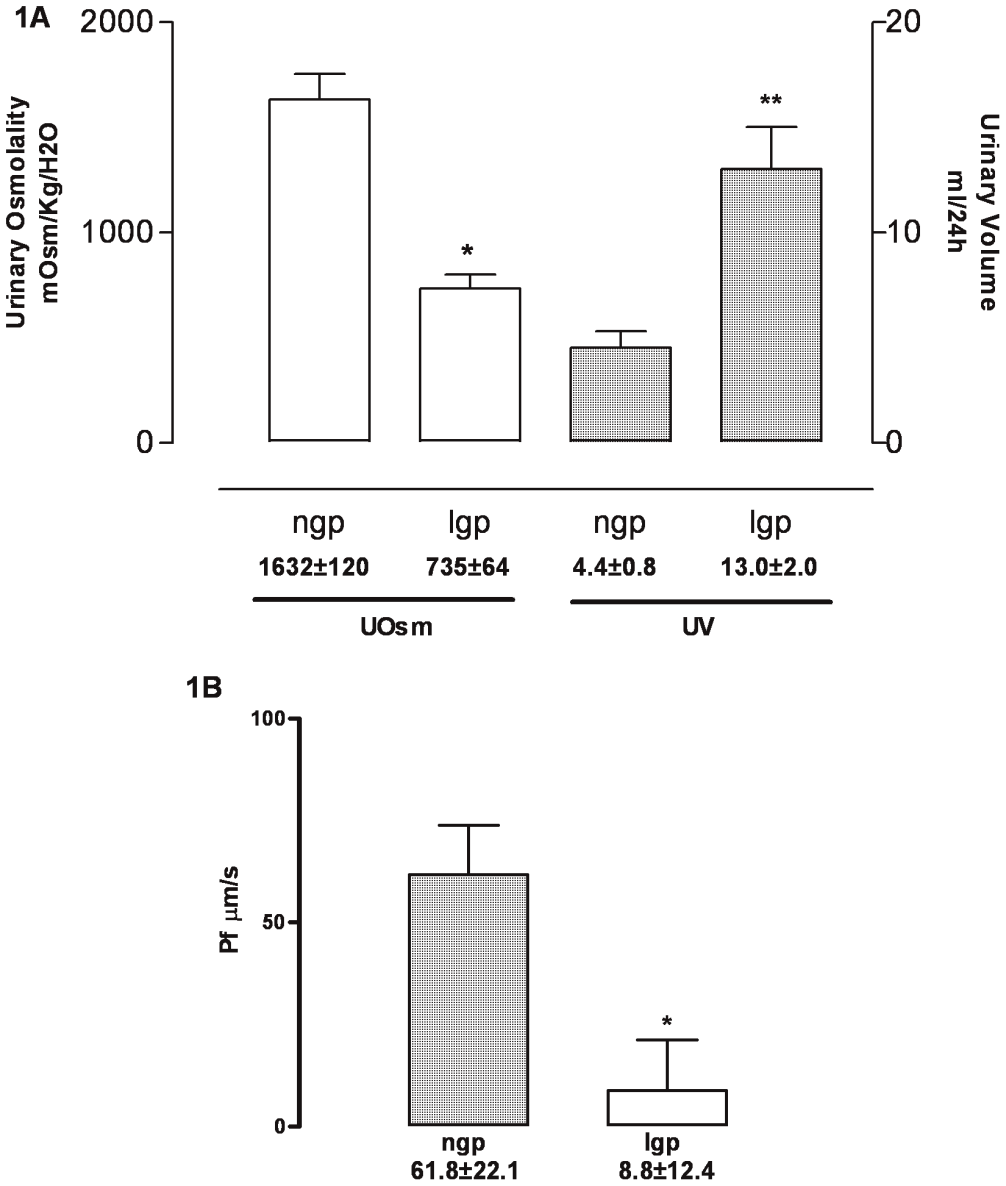
Leptospirotic guinea pig data. A- Urinary Volume (ml) and Urinary Osmolality (mOsm/Kg H_2_O). Dotted bar-UOsm; Open bar-UV. B- Water permeability. Pf µm/s. Values are expressed as mean ± SEM. Significant differences: * p<0.01; ** p<0.05.

In the first group of experiments, normal guinea pigs (ngp, n = 5) were compared with the leptospirotic guinea pigs (lgp, n = 5). In the leptospiroric animals, the urinary volume (milliliters) and the BUN (mg %) were higher than the normal guinea pigs, but the urinary osmolality (mOsm/Kg) and the osmotic water permeability (Pf µm/s) were lower (Table 1, fig. 1A and 1B). These results evidenced that the infected guinea pigs presented urinary concentration incapacity and lower osmotic water permeability in comparison with normal guinea pigs.

**Table 1:** Data from normal and leptospirotic guinea pigs.

N	Pf	UV	UOsm	BUN
ngp 5	61.8±22.1	4,5±0.8	1632±120	56±9
lgp 5	8.8±12.4*	13.0±2.0**	735±64*	178±32**

Values are expressed as mean±SEM. Pf- Osmotic Water Permeability, µm/s; UOsm- Urinary Osmolality, mOsm/Kg H_2_O; UV- Urinary Volume ml; BUN-Blood Urea Nitrogen mg%; ngp- normal and lgp leptospirotic guinea pigs.

** -p<0.05;

* p<0.01 vs ngp values.

### Group II - Normal Animals

In the second group of experiments (n = 54), in order to determine the point at which the glycolipoprotein was acting, Pfs from normal guinea pigs IMCDs, in the presence of 250 µg/ml GLP and in the presence of activators of various steps of vasopressin cascade, were determined. In this group, GLPc was added to the bath solution, in one set of experiments before and in another set after the drugs were assayed.

#### GLPc effect on vasopressin action

**Figure 2:**
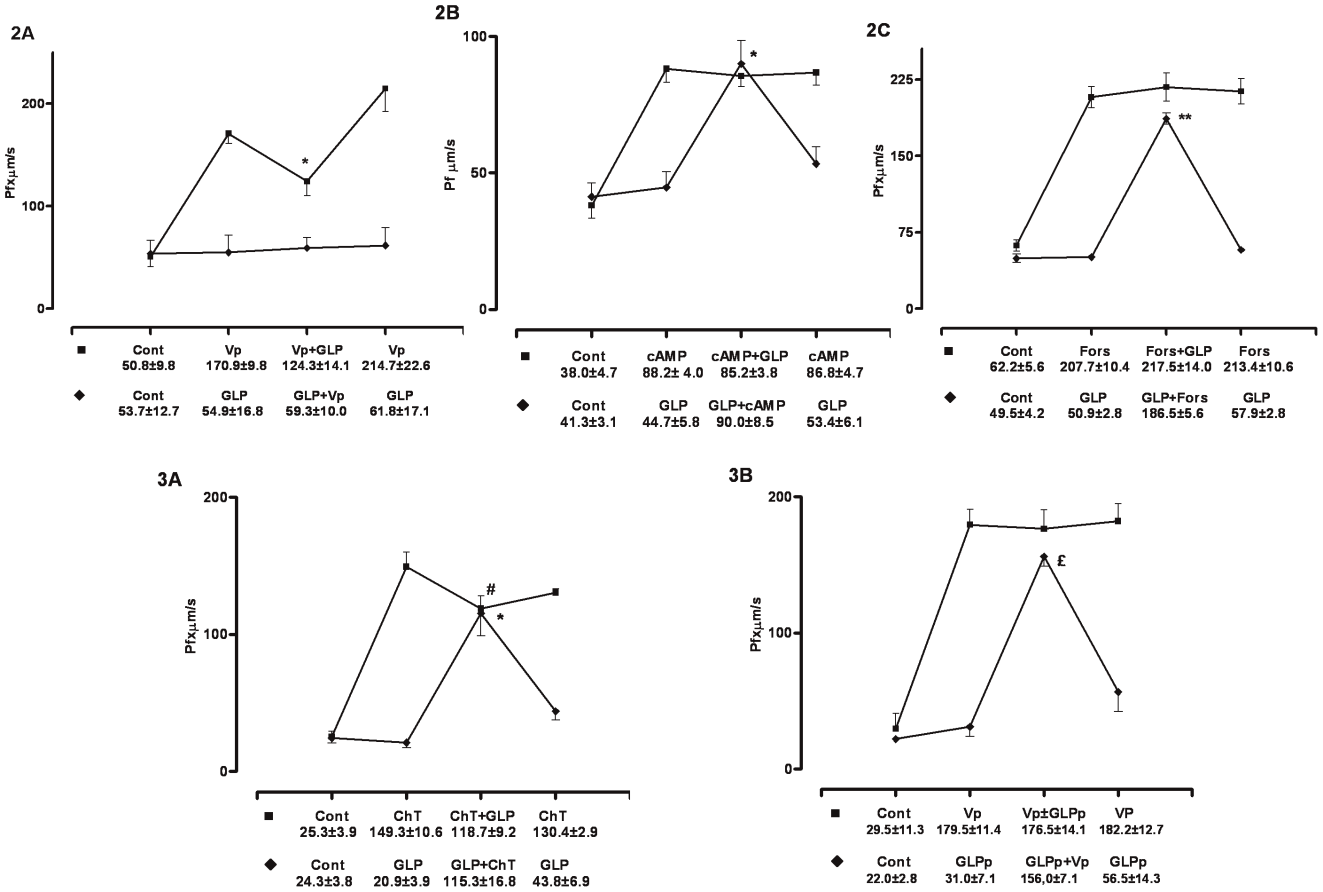
IMCD water permeability. Effects of GLPc (250 µg/ml) on: 2A - Vasopressin action (Vp, 200 pg/ml, n = 5), 2B - cAMP activity (10^−5^ M), 2C - Forskolin action (Fors 10^−9^ M, n = 5), 2D - Cholera Toxin action (ChT 10^−9^ M, n = 6), 2E - effect of GLPp on Vp action (GLPp- 250 µg/ml, n = 5). Values are expressed as mean ± SEM. Lines connect the averages of the data for each different period of the experiments. Significant differences: * p<0.02, **p<0.01. £ p<0.05, # p<0.001.

In these experiments the effect of GLPc on Vp ([Arg^8^] Vasopressin, 200 pg/ml) action was studied. First, GLPc was applied to the bath solution after Vp (n = 5) and then, in other experiments, the GLPc was added before the addition of Vp (n = 6). As the data showed, GLPc partially blocked the Vp-stimulated Pf when applied after Vp and practically blocked all the Vp action when applied before Vp. (Table 2, and Figure 2A).

**Table 2:** Data from microperfusion experiments - Pf µm/s.

n = 5	Control 50.8±9.8	Vp 170.9±9.8	Vp+ GLPc 124.3±14.1*	Vp 214.7±22.6
n = 6	Control 53.7±12.7	GLPc 54.9±16.8	GLPc+Vp 59.3±10.0	GLPc 61.8±17.1
n = 5	Control 41.3±3.1	GLPc 44.7±5.8	GLPc+cAMP 90.0±8.5 *	GLPc 53.4±6.1
n = 5	Control 38.0±4.7	cAMP+GLPc 88.2±4.0	GLPc 85.2±3.8	cAMP+GLPc 86.8±4.7
n = 6	Control 49.5±4.2	GLPc 50.9±2.8	GLPc+Fors 186.5±5.6**	GLPc 57.9±2.8
n = 5	Control 62.2±5.6	Fors 207.7±10.4	Fors+GLPc 217.5±14.0	Fors 213.4±10.6
n = 6	Control 25.3±3.9	ChT 149.3±10.6	ChT+GLPc 118.7±9.2#	ChT 130.4±2.9
n = 6	Control 22±2.8	GLPc 20.9±3.9	GLPc+ChT 115.3±16.8*	GLPc 43.8±6.9
n = 5	Control 22±2.8	GLPp 31.0±7.1	GLPp+Vp* 156±7.1£	GLPp 56.5±14.3
n = 5	Control 29.5±11.3	Vp 179.5±11.4	Vp+GLPp 176.5±14.1	Vp 182.2±12.7

The cells in each row, from left to right, represent successive experimental periods. Values expressed in mean+SEM. Mean Vi, 30.9±0.08 nl/min; mean area 15.7±1.0×10^−4^ cm^2^ Vp-Vasopressin; Fors-Forskolin; ChT-Cholera Toxin;

* p<0.02;

** p<0.01;

# p<0.05;

£ p<0.001, all vs the preceeding periods.

#### GLPc effect on cAMP action

The effect of GLPc on cAMP (N^6^-2′-O-Dibutyryladenosine 3′ 5′cyclic Monophosphate 10^−4^ M, n = 5) was studied in the absence of Vp. These experiments showed that the cAMP stimulated-Pf was not blocked by GLPc, evidencing that the GLPc effect is before AMPc generation. (Table 2, Figure 2B).

#### GLPc effect on forskolin action

Forskolin (Fors 10^−9^ M) is a stimulator of the adenylyl cyclase and is able to increase the Pf in the absence of Vp. The GLPc did not block the forskolin stimulated-Pf (n = 5). This non-blocking forskolin effect demonstrated that GLPc was not interfering in the adenylyl cyclase function (Table 2, Figure 2C).

#### GLPc effect on cholera toxin effect

Cholera Toxin (ChT 10^−9^ M) is a stimulator of Protein G and can elicit the Vp cascade from this point on. In the absence of Vp, these experiments (n = 6) showed that GLPc was able to partially decrease the ChT effect from 149.3±11.0 to 118.7±9.2, providing evidence that this toxin is decreasing the action of protein G complex. The Pf stimulated by ChT (149.3±11.0) and the Pf in the presence of GLPc+ChT (115.3±16.8) were also significantly different (p<0.02), but the values 118.7±9.2 (ChT+GLPc) and 115.3±16.8 were statistically similar (Table 2, Figure 2D). These data showed that the Cholera toxin was partially blocked by the GLP action.

#### GLP patoc effect on vasopressin action

GLP patoc (GLPp- 250 µg/ml) was extracted from a non-pathogenic *Leptospira* and was used as a control of the pathogenic GLP. The data showed that the vasopressin stimulated-Pf (n = 5) was not blocked by GLPp. (Table 2, Figure 2E).

### Group III - GLP Animals- Western Blot Study

**Figure 3:**
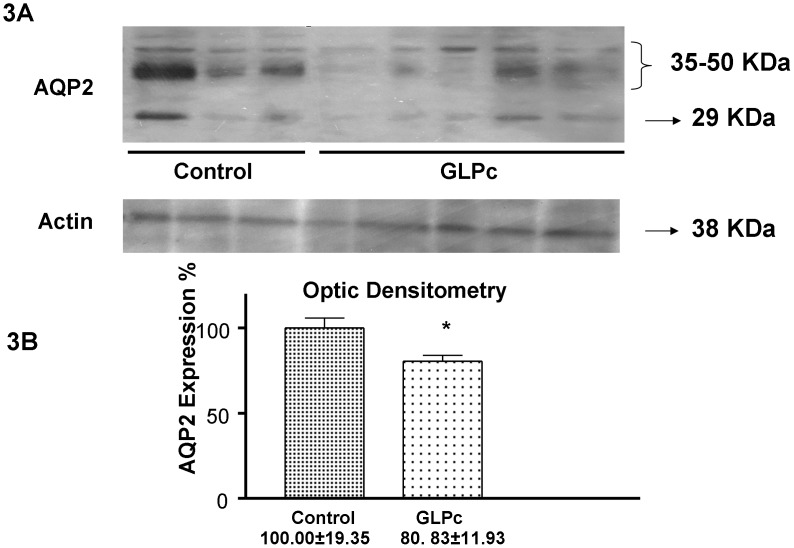
Western blot analysis of water transporter protein in IMCD from normal (n = 3) and injected i.p. with GLPc (n = 5) guinea pigs. 3A- Western blot analysis of AQP2 protein expression showing the 29 KDa and 35–50 KDa bands and the actin band; 3B- Quantitative densitometric analysis of AQP2 protein abundance. * p<0.05 vs. control.

Immunoblotting studies showed that both the 29-kDa and the 35–50 kDa bands were less expressed in infected guinea pigs than in normal ones (Figure 3A). The densitometric analysis of the immunoblotting assays showed a decrease in AQP2 protein abundance of approximately 20% compared to the control: 100.00±5.92 (n = 3) *vs.* GLP 79.83±3.53 (p<0.05) (n = 5) (Figure 3B).

## Discussion

*Leptospira* is a genus divided into pathogenic strains, such as *L. interrogans,* and saprophytic strains, such as the free-living *L. biflexa*, species [15]. Only the pathogenic species cause leptospirosis. In 1968, Stalhein [16] reported that lipids extracted from leptospira were toxic to guinea pig macrophages *in vitro*, and Vinh et al [12] showed that glycolipoproteins from leptospira culture produced a cytotoxic effect on fibroblast cells in culture. In recent years, several studies have been carried out to investigate the immunologic and inflammatory alterations in leptospirosis as well as to determine the genome sequence of the *Leptospira* [17]–[20].

In a previous study, it had been already proposed that this concentration defect observed in leptospirotic acute renal injury could be explained, at least in part, by a decrease in vasopressin responsiveness due to a primary cellular defect [7].

In Group I, the infected animals had an increase in BUN concentration as a consequence of the glomerular filtration rate reduction caused by the severe leptospirosis infection. The increase of the urinary volume and the decrease of the urinary osmolality are a consequence of the urinary concentration defect. This concentration defect was explained by the very low water permeability in the infected guinea pigs in comparison with the normal animals. These data are in accordance with those found in leptospirotic patients with acute renal injury.

In Group II, experiments were carried out in order to investigate if GLP could be one of the toxins responsible by this defect. The direct effect of GLPc on IMCDs from normal guinea pigs was studied. In the first GLPc experiment, when this endotoxin was applied to the bath after vasopressin, the hormone effect dropped about 30%, but when GLPc was applied before, it blocked the hormone effect completely. These results could be interpreted as evidence that the GLPc is acting on the Vp cascade and these different data between the two experiments could also be interpreted as evidence that the GLPc is competing with the Vp for the Vp receptor. Although this is an attractive hypothesis, binding experiments would have to be carried out to prove it.

In order to study at which point of the vasopressin cascade this cytotoxin is acting, initially the GLPc effect on the action of cAMP was analyzed. GLPc did not block cAMP, indicating that it acted before cAMP synthesis. To verify the GLPc effect on adenylyl cyclase, forskolin, an adenylyl cyclase stimulator, was used. The results demonstrated that GLPc did not decrease the forskolin effect, indicating that in this step of the vasopressin cascade, GLPc is not acting either. To study the GLPc effect on protein G (guanine nucleotide-binding protein), cholera toxin was used (a stimulator of the protein-G system). Its effect was partially inhibited (about 15%) by GLPc, demonstrating that the L*eptospira* endotoxin is acting on the protein-G system. A GLP extracted from a non-pathogenic *Leptospira* (*Leptospira biflexa* serovar Patoc strain Patoc I, extracted by the same procedure for extracting the pathogenic GLP), was used as a control of the pathologenic GLP. The results demonstrated that this GLPp was not able to block the effect of vasopressin, showing that the substances used in the GLP extraction did not interfere in its action (see MM).

The substantial reduction of the Pf, observed in infected guinea pigs, could be undoubtedly caused not only by the GLP effect but also by the effects of the large quantity of other toxins and inflammation mediators released into the blood stream due to the severe systemic infection. In the microperfusion experiments in the second Group the conditions were substantially different, where the amount of GLPc used was, certainly, much lower than that in the blood stream in the infected animals. Even so, the Pf was decreased, showing its effect evidencing that the GLP is acting on the Vp-Protein G complex.

In order to study the effect of GLPc on the expression of Aquaporin 2, a third group was included. The procedure chosen was to inoculate GLPc by the intraperitoneal route in normal guinea pigs, the same procedure used to infect the other animals. This experiment showed that the AQP2 expression decreased about 20%, indicating that GLP could also act when applied into the peritoneal cavity. Although 20% is a small decrease in comparison to the infected animals, this amount is enough to promote an increase in the urinary volume.

Renal involvement in leptospirosis is well documented, but the effect of GLP on renal function is rarely reported [3], [6], [7], [21], [22], [23]. Santos et al [21], using an antibody against GLP, demonstrated its distribution in the kidneys and livers of infected guinea pigs. Pereira et al [24] showed that leptospiral antigens (GLP) bind and cause damage to the proximal and distal segments of the nephron. These lesions on proximal and distal tubules can explain the natriuresis, hypokalemia, kaliuresis and the concentrating defect, as was reported by Seguro et al [5] in patients and demonstrated by Magaldi et al [7] in experimental studies.

The polyuria observed in leptospirosis could be explained by at least two mechanisms: 1- Proximal tubule injury - it is well known that animals with leptospirostic renal insufficiency present focal interstitial nephrites [3], [25] and an impairment in the concentrating process, accompanied by natriuresis, kaliuresis and hypokalemia [5], [7], whose origins were recently and elegantly demonstrated by Andrade et al [4] and Araujo et al [26,]. In these last studies, a decrease in the NHE3 protein abundance was demonstrated. This decrease in the NHE3 function promotes low sodium reabsorption and, consequently, low water reabsorption thus increasing the delivery of sodium and water to the distal nephron, which in turn causes natriuresis and contributes to the increase in urinary volume. 2- Decreased vasopressin responsiveness in IMCD - this fact is accountable for the decrease in AQP2 expression in IMCDs, shown in the present study in guinea pigs inoculated with GLP and also demonstrated by Andrade et al [4] in infected hamsters. This occurrence can explain the decrease in water absorption in this segment, resulting in an inability to concentrate urine and an increase in urinary volume. Another possibility could be considered taking into account studies showing the inhibitory effect on Na,K ATPase by GLP and on Na, K, Cl co-transporter NKCC2 by an outer membrane protein, which could contribute to a decrease in the formation of the medullary hypertonicity [27], [28]. Moreover, it is well known that hypocalemia can cause a decrease in the Vp action leading to a decrease in water absorption. However, in our *in vitro* experiments the potassium levels remained constant during the perfusion.

The recovery of the renal function after AKI is quickly, but in some cases a urinary concentration incapacity remains for more than six months [29].These findings could reveal that the tubular injuries in the AKI were very severe and did not have good recovery.

On the other hand, in direct opposition to our data, Araujo et al [26] using immunohistochemistry, detected an increase instead of a decrease in AQP2 expression in collecting tubules from both patients with acute kidney injury caused by leptospirosis and by acute tubular necrosis caused by other pathologies. This finding was interpreted by the authors as a compensatory response to retain the excess of water delivery to the distal nephrons in a renal recovery phase of the disease, since in these two pathologies,the proximal tubules were damaged. However, these data were obtained from autopsies and depended on the severity and on the stage of the disease at the time of patients’ death, while our data were obtained from infected animals whose sacrifice was programmed and in an initial phase of the disease.

Taken as a whole, our data showed that GLPc applied on isolated IMCDs from normal guinea pigs, could reproduce the results found in experiments with guinea pigs infected with the spirochaetes providing evidences that this endotoxin is an important contributor to the virulence of the *Leptospira*.

Finally, the urinary concentration defect observed in patients with leptospirosis can be explained, at least in part, by the decrease of vasopressin responsiveness, which induces a decrease in water absorption in the last nephron segment.
